# Translation and validation of the Malay version of Shiffman-Jarvik withdrawal scale and cessation self-efficacy questionnaire: a review of psychometric properties

**DOI:** 10.1186/s12955-015-0238-0

**Published:** 2015-04-09

**Authors:** Eng Wah Teo, Yuin Yi Lee, Selina Khoo, Tony Morris

**Affiliations:** Sports Centre, University of Malaya, Kuala Lumpur, Malaysia; Institute of Sport, Exercise and Active Living (ISEAL), College of Sport and Exercise Science, Victoria University, Melbourne, Australia

**Keywords:** Smoking cessation, Cultural adaptation, Reliability, Malaysia, CSEQ, SJWS

## Abstract

**Background:**

Smoking tobacco is a major concern in Malaysia, with 23.1% of Malaysian adults smoking tobacco in 2012. Withdrawal symptoms and self-efficacy to quit smoking have been shown to have significant effects on the outcomes of smoking cessation. The Shiffman-Jarvik Withdrawal Scale (Psychopharmacology, 50: 35-39, 1976) and the Cessation Self-Efficacy Questionnaire (Cognitive Ther Res 5: 175-187, 1981) are two questionnaires that have been widely used in various smoking cessation research. The short SJWS consists of 15 items with five subscales: physical symptoms, psychological symptoms, stimulation/sedation, appetite, and cravings. The CSEQ is a 12-item questionnaire that assesses participant’s self-efficacy to avoid smoking in various situations described in each item. The aim of this study was to translate and validate the Malay language version of the SJWS and the CSEQ.

**Methods:**

The SJWS and CSEQ were translated into the Malay language based on the back translation method. A total of 146 participants (25.08 ± 5.19 years) answered the translated questionnaires. Psychometrics properties such as reliability (internal consistency and test-retest) and validity (content validity, construct validity and face validity) were examined.

**Results:**

Both questionnaires showed acceptable internal consistency; SJWS-M (α = 0.66) and CSEQ-M (α = 0.90) and good test-retest reliability; SJWS-M (r = 0.76) and the CSEQ-M (r = 0.80). SJWS-M (χ^2^ = 15.964, GFI = 0.979, CFI = 1.000, RMSEA = 0.000, ChiSq/df = 0.939, AGFI = 0.933, TLI = 1.004, and NPI = 0.978) and CSEQ-M (of χ^2^ = 35.16, GFI = 0.960, CFI = 0.999, RMSEA = 0.015, ChiSq/df = 1.034, AGFI = 0.908, TLI = 0.999, and NPI = 0.979) also showed good construct validity. Both questionnaires showed sufficient item to item convergent validity and item discriminant validity. Content validity was established (reassess) by experts in the field of psychology, culture and language whereas face validity was confirmed by smokers.

**Conclusions:**

The translated Malay version of the CSEQ-M and the SJWS-M showed great reliability and validity evidences therefore is an adequate and useful instrument to evaluate Malaysian smokers. Future studies could investigate differences in self-esteem between long-term and short-term smokers and evaluate the usability of these questionnaires in local smoking research and other Malay speaking countries (Brunei and Indonesia).

## Background

Smoking is a global concern with approximately 1.1 billion smokers worldwide [1]. Tobacco abuse is one of the leading risk factors for mortality globally, responsible for 12% of all deaths in adults aged 30 years and over [2]. In addition, previous literature has shown that smokers and ex-smokers have lower quality of life compared to non-smokers such as lower physical functioning, social functioning, general health and mental health [3-6].

Smoking is a major concern in Malaysia. The Institute for Public Health reported in 2012 that 23.1% of Malaysian adults smoke [7]. In 2010, smoking was ranked one of the top three leading risk factors in Malaysia and accounted for diseases such as cardiovascular and circulatory diseases, chronic respiratory diseases, and cancer [8]. According to the Global Adult Tobacco Survey, 88.1% of current Malaysian smokers surveyed in 2011 believed that smoking causes serious illness [7]. Despite the detrimental effects of smoking, many Malaysians still continue to smoke. Thus, there is an urgent need for improved strategies and programs to combat smoking addiction.

The addictive nature of various chemicals found in cigarettes continuously makes smoking cessation difficult [9,10]. Studies have been conducted on various aspects of smoking cessation including predictors of smoking, effects of smoking, interventions in smoking cessation, withdrawal symptoms, and smoking cessation self-efficacy [1,11-15]. Researchers have used theories of health behavior, including Bandura’s Self-Efficacy Theory [16] and Ajzen’s Theory of Planned Behavior [17] to explain smoking behaviors and identify the factors associated with smoking cessation outcomes [13,18,19]. These theories emphasized individuals’ self-confidence in their own ability to perform a certain task. According to self-efficacy theories, efficacy expectations can predict the degree of effort and persistence an individual will exert in order to achieve one’s goal [16,17]. In relation to smoking, these theories suggest that individuals with low cessation self-efficacy will invest little effort to avoid smoking or maintain smoking abstinence, compared to individuals with higher levels of cessation self-efficacy. These theories are supported by studies conducted on smoking cessation related self-efficacy [12,13,20]. These studies revealed that the readiness to quit and high cessation related self-efficacy often predict lower levels of withdrawal symptoms throughout the cessation process and higher likelihood of abstinence after completing a smoking cessation program.

Another area that has been widely studied is withdrawal symptoms among smokers undergoing smoking cessation [13,21,22]. Previous literature has identified several smoking abstinence withdrawal symptoms which include craving, anger or irritability, anxiety, depression, difficulty concentrating, drowsiness and impatience [21,23]. Swan and colleagues [14] studied withdrawal symptoms as predictors of relapse and found that anger, depressed mood and craving are strong predictors of relapse within one month of abstinence. This finding is supported by other research on withdrawal and smoking cessation outcomes [24,25].

Cessation-related self-efficacy and withdrawal symptoms during abstinence strongly affect smoking cessation outcomes [13]. Studying the possible interactions between these two factors could be important in understanding nicotine dependence and improving smoking cessation programs. According to Bandura’s Self-Efficacy Theory, negative emotional arousal may affect individuals’ self-efficacy in coping with threatening situations [16]. In the case of smoking cessation, withdrawal symptoms such as irritability, anxiety, depression and drowsiness may affect smokers’ self-efficacy to quit smoking or maintain abstinence during or after the cessation process. A study by Morrell and colleagues suggested that while high levels of withdrawal symptoms did not predict low levels of self-efficacy, low levels of self-efficacy after 24 hours of abstinence predicted high levels of withdrawal symptoms after 48 hours of abstinence [13]. They suggested that smokers with low self-efficacy might have negative expectancies about smoking cessation, which in turn heighten their sensitivity towards withdrawal symptoms. While this provides great insight towards the link between self-efficacy and withdrawal symptoms, further research is needed to provide more comprehensive findings.

In order to collect accurate data on smoking cessation self-efficacy and withdrawal symptoms among smokers in Malaysia, valid and reliable measures are required. Although such measures have been developed and validated worldwide, most of them have not been culturally and linguistically adapted for use in Malaysia. Limited effort has been made to translate these questionnaires into valid and reliable Malay language questionnaires [26,27]. Many smoking cessation studies in Malaysia have measured levels of nicotine dependence, rather than withdrawal symptoms [12,28,29]. Based on the current literature, only one study in Malaysia focused on withdrawal symptoms. The researchers in that study translated the Wisconsin Smoking Withdrawal Scale to Malay language [26]. Ideally, researchers should measure nicotine dependence and withdrawal to establish a more complete picture. We consider the SJWS to be a useful measure of withdrawal, so we translated the SJWS into Malay for use in our research and in future studies in Malaysia. No study in Malaysia has measured smoking cessation self-efficacy comprehensively using a questionnaire. It is important to understand the role of confidence in smokers’ ability to stop smoking, in the smoking cessation process. Thus, we translated the CSES into Malay for use in our research and future studies in Malaysia. The aim of this study was to translate and validate the Malay versions of cessation-related self-efficacy and withdrawal symptoms questionnaires. We focus on two commonly used questionnaires for smoking cessation, namely the Shiffman-Jarvik Withdrawal Scale (SJWS) [21] and the Cessation Self-Efficacy Questionnaire (CSEQ) [30]. Both questionnaires are widely used in smoking cessation studies [31-35].

## Methods

### Participants

A convenience sample of 146 smokers was recruited from the states of Selangor, Kuala Lumpur and Putrajaya. Participants were between 18–65 years old and currently not receiving any psychiatric and drug treatment. In this study, smokers were those with smoking history of three or more cigarettes a day for at least two years. All data analyses were based on 146 participants, however for test-retest reliability only 79 out of 146 participated, while a separate 10 participants were recruited for face validity during the end of the questionnaire translation process. The participants for this study were 98% male and 2% female with mean age of 25.08 ± 5.19 years. Age groups of participants are divided to < 20 years (0.7%), 21–30 years (64.4%), 31–40 (21.2%), 41–50 (5.5%) and 51–60 (7.5%) and > 60 years (0.7%). The majority of the participants were predominantly Malay (60.3%) followed by Chinese (19.9%), Indians (17.8%) and other (2.1%). Smoking history ranged from 0.33 - 42 years (*M* = 13.06 ± 9.9 years). Prior to data collection, participants were briefed on the purpose and nature of the study, and informed consent was obtained from participant. This study was approved by the Ministry of Health Medical Research Ethics Committee (NMRR-13-159-15234).

### Measures

The Shiffman-Jarvik Withdrawal Scale (SJWS) 15-item version has been used in smoking cessation research and demonstrated sound internal consistency with Cronbach’s alpha, α = 0.76 - 0.82 [32,36]. In this study, we translated and validated the modified 15-item version of the SJWS. The SJWS is a 25-item questionnaire abstracted from the original 43-item questionnaire developed by Gritz and Jarvik in 1973 [37]. The questionnaire assesses participants’ desires to smoke as well as other withdrawal symptoms that may occur such as inability to sleep, anxiety and inability to concentrate. The questionnaire consists of five subscales: craving (five items), psychological symptoms (five items), physical symptoms (three items), sedation (one item) and appetite (one item). “If you could smoke freely, would you like a cigarette this minute?” is an example of an item from the cravings subscale, “Do you feel content?” from the psychological symptoms subscale, “Do you have fluttery feelings in your chest right now?” from the physical symptoms subscale, “Do you feel wide awake?” from the sedation subscale and “Is your appetite smaller than normal?” from the appetite subscale. Participants choose their responses from a 7-point Likert scale, with responses ranging from “very definitely” to “very definitely not”. While this questionnaire tends to result in reliable scales because items are grouped according to their inter-correlations, it was developed before nicotine withdrawal was defined more clearly [35]. Items in the original scale that were not considered a core part of the current definition of nicotine withdrawal (i.e., somatic symptoms) were deleted resulting in a shorter 15-item version [35].

The CSEQ was developed by Diclemente [30] as a measure of smokers’ self-efficacy for avoiding smoking in various situations are described in 12 items. In the original study, these situations were identified by subjects as important factors in relapse episodes. Examples of items are “When alone and feeling depressed”, “Over coffee while talking and relaxing”, and “When I see that I am gaining weight”. Participants respond using a 7-point Likert scale, with responses ranging from “completely unsure” to “completely sure”. Pearson item correlation was reported at an average of 0.68 ranging from 0.58 to 0.76 [30]. The CSEQ showed sound internal consistency with α of 0.87 [31] and 0.96 [34].

### Translation and cultural adaptation

We employed the back translation procedure based on Brislin’s model [38] for our cross-cultural smoking study. Five bilingual translators (fluent in both Malay and English) with at least 3–5 years of translation experience were recruited. Translators were Malay-native speakers and received English language education from primary school to university. The translation was based on “ask the same question and provide the same response options” [39]. It was explained that in a general survey research, translations of questionnaires should ask the same questions and offer the same response as the source questionnaires. This is expected to be achieved by translating the source questionnaires as closely as possible. Close translation of questionnaires was conducted by using words that were as close as possible in sense and meaning to the meaning of items referred to in the original questionnaires [39]. The aim of the translation was to achieve conceptual equivalence in addition to literal or syntactic equivalence.

The first group of translators (three translators) independently translated the questionnaires from the source language (English) into the target language (Malay). The research team compiled and compared all three translations to create the Malay version. A second group of bilingual translators blindly (without referring to the original version) and independently back translated the Malay version to English [40]. The research team reviewed and evaluated both English versions (Malay to English version vs. original English version). Item discrepancies, language difficulty, clarity, and possible cultural insensitivity were evaluated and rectified by the research team. Iteration from English to Malay and then back to English was conducted. After two iterations, the research team was satisfied with the final Malay version. The final Malay version was then tested for face validity.

### Data analyses

The Statistical Package for Social Science (SPSS), version 17.0 [41] was used for data analyses. Kolmogorov-Smirnov, skewness, and kurtosis tests were employed to check for data normality. Data were shown to be normally distributed. The AMOS version 21 software was used for Confirmatory Factor Analysis (CFA) and Structural Equation Modeling (SEM).

#### Reliability

According to Hendrickson, Massey and Cronan [42], three popular reliability tests are Cronbach’s alpha (internal consistency), replication with different samples, and test-retest (stability). The internal consistency indicates the degree to which a set of items measures a single uni-dimensional latent construct [43]. In other words, it measures whether a group of items that was supposed to evaluate the same factor or subscale produces similar or almost similar scores [44].

Test-retest examines item stability using the same measures administered to participants at two different occasions within a chosen time period. The test-retest procedure has been applied to many culturally- and linguistically-translated instruments [45-47]. Allen and Yen [48] explained that using a very short time interval between tests could cause carryover effects due to memory, practice, or mood. On the other hand, a longer time interval increases the chances for a change in status. In our present study, 79 participants were recruited for the test-retest procedure. Participants answered both questionnaires (SJWS-M and CSEQ-M) and then re-answered them under similar conditions after a 2-week interval.

#### Validity

Various validity tests were conducted namely content validity, construct validity, and face validity. Content validity was examined during the translation process by bilingual translators and then checked by a cultural specialist, language specialist, and sport psychologist (research team members) for content conformity and culture bias at the end of the translation process. Later, ten random smokers reconfirmed the final version of the SJWS-M and the CSEQ-M questionnaires for face validity tests. Both questionnaires include a comment section for each question to check for face validity.

Construct validity: Confirmatory Factor Analysis using Structural Equation Modeling (SEM) was performed to assess model fit using the AMOS 21 software. Hair and colleagues [49] suggested at least four fit indexes to estimate construct validity of a measurement model: Chi Square/degree of freedom (Chisq/df), Goodness of Fit Index (GFI), Comparative Fit Index (CFI), and Root Mean Square of Error Approximation (RMSEA). The larger the probability associated with the chi-square, the better the fit of the model to the data [50]. The GFI test is the goodness of fit index and ideally should be greater than 0.90 to reflect a good fit. The RMSEA is a population based index and consequently is insensitive to sample size. A RMSEA of < 0.10 is considered good and < 0.05 is very good [50], although Hu and Bentler [51] recommended a RMSEA of < 0.06. Convergent, discriminant, and construct validity were also obtained based on the SEM analysis [52].

## Results

### Translation and cultural adaptation

Through iterations of the translation process, we found a minor cultural bias in Item 8 of the CSEQ (“While drinking in bar”). Although this item is relevant in the smoking behavior framework, it was deemed inappropriate for Muslims participants who make up 61.3% of the Malaysian population [53]. Muslims are prohibited from visiting bars or consuming alcohol. Hence, this item was deemed insensitive towards Muslim respondents and as a result the terminology “bar” was later changed to “party” based on cultural and religious grounds. All other items were considered to be culturally appropriate, easy to understand and achieved conceptual equivalence.

### Reliability evidence

Overall Cronbach’s alpha (α) values for the SJWS-M questionnaire was α = 0.66, with subscale Craving (4 items), α = 0.89, Physical symptoms (3 items), α = 0.73, and Psychological symptoms (3 items), α = 0.79. However, two other subscales Stimulation/ Sedation (1 item) and Appetite (1 item) were not analyzed because the number of items was less than three or four items. While the 12-item CSEQ-M questionnaire with no subscale revealed an overall Cronbach’s alpha of 0.90 (Table 1). Hence, both questionnaires demonstrated sufficient internal consistency [54,55].

**Table 1:** **Cronbach’s alpha**

	**Cronbach’s alpha (α)**	**Number of item**
SJWS-M (prior to CFA)		
Overall	0.75	15
SJWS-M		
Overall	0.66	10
Craving	0.89	4
Physical symptoms	0.73	3
Psychological symptoms	0.79	3
Stimulation/Sedation	not included	1
Appetite	not included	1
CSEQ-M		
Overall	0.90	12

Test-retest reliability was checked based on Pearson’s product–moment correlation using 79 test-retest participants. The SJWS-M test-retest yielded a correlation of r(79) = 0.76, whereas the CSEQ-M yielded correlation of r(79) = 0.80, indicating good test-retest reliability for the Malay versions of both questionnaires (Table 2).

**Table 2:** **Test-retest**

	**Correlations (** ***r*** **)**
SJWS-M	0.76
CSEQ-M	0.80

### Validity evidence

**Figure 1:**
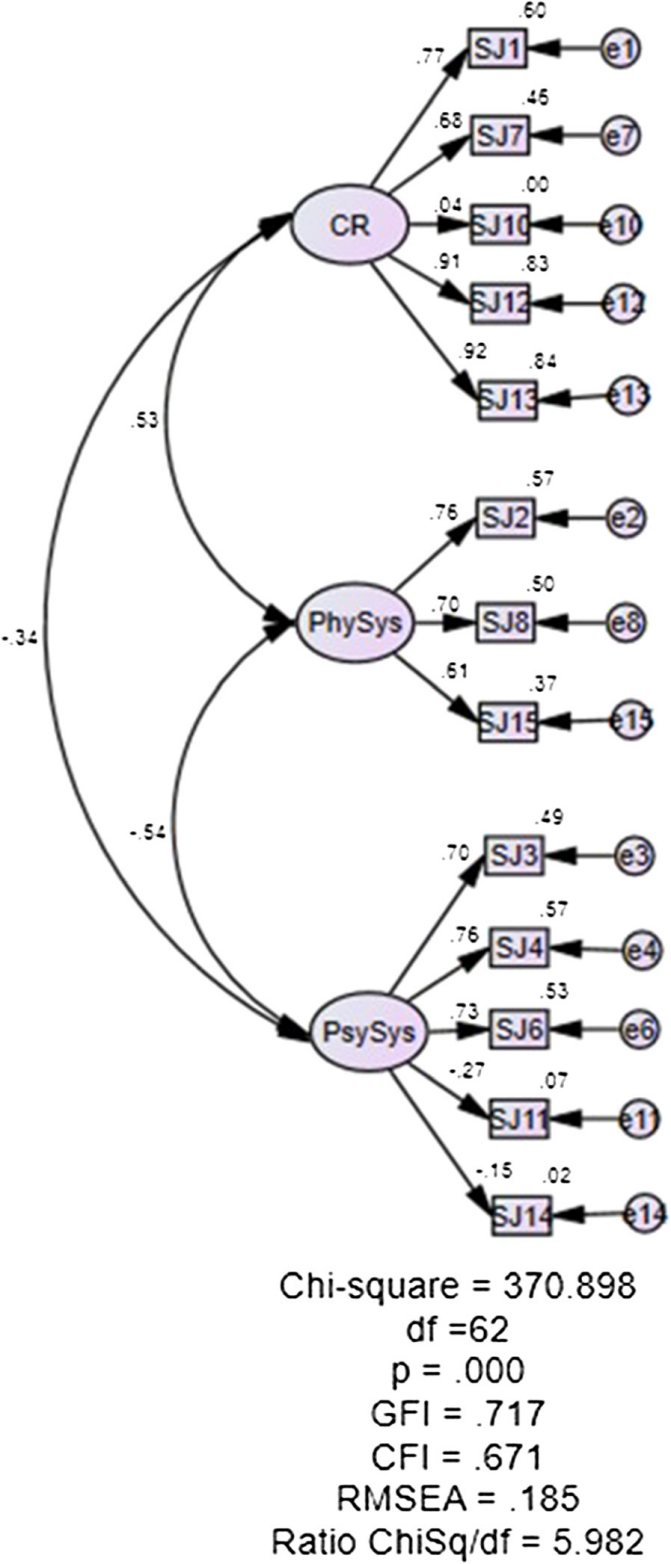
**Initial factor loading for SJWS-M (deletion of item 10, 11 14).**

Based on Structural Equation Modeling (SEM), two items from the SJWS-M were not included because subscale Stimulation/Sedation and Appetite had only one item each (Items 5 and Item 9). Another three items (Items 10, Item 11 and Item 14) were deleted due to factor loadings that are lower than 0.50 (Figure 1). Hence, the total items were reduced from the original 15 items (SJWS) to 10 items (SJWS-M). While no items deletion was performed on CSEQ-M as all factor loadings were above 0.50. Based on the CFA and SEM analysis, various other validity evidence were also obtained as shown below.

Item to item convergent validity was checked based on proposed covariance values CR (critical values) of more than ± 1.96, *p* < 0. 05. The majority of covariance CR values for SJWS-M and CSEQ-M were above the suggested value except for five items pairing for SJWS-M and seven items pairing for CSEQ-M. Discriminant validity for items was tested using item correlation, which showed that there were no redundant items, as correlations between each pair of items were less than 0.85.

**Figure 2:**
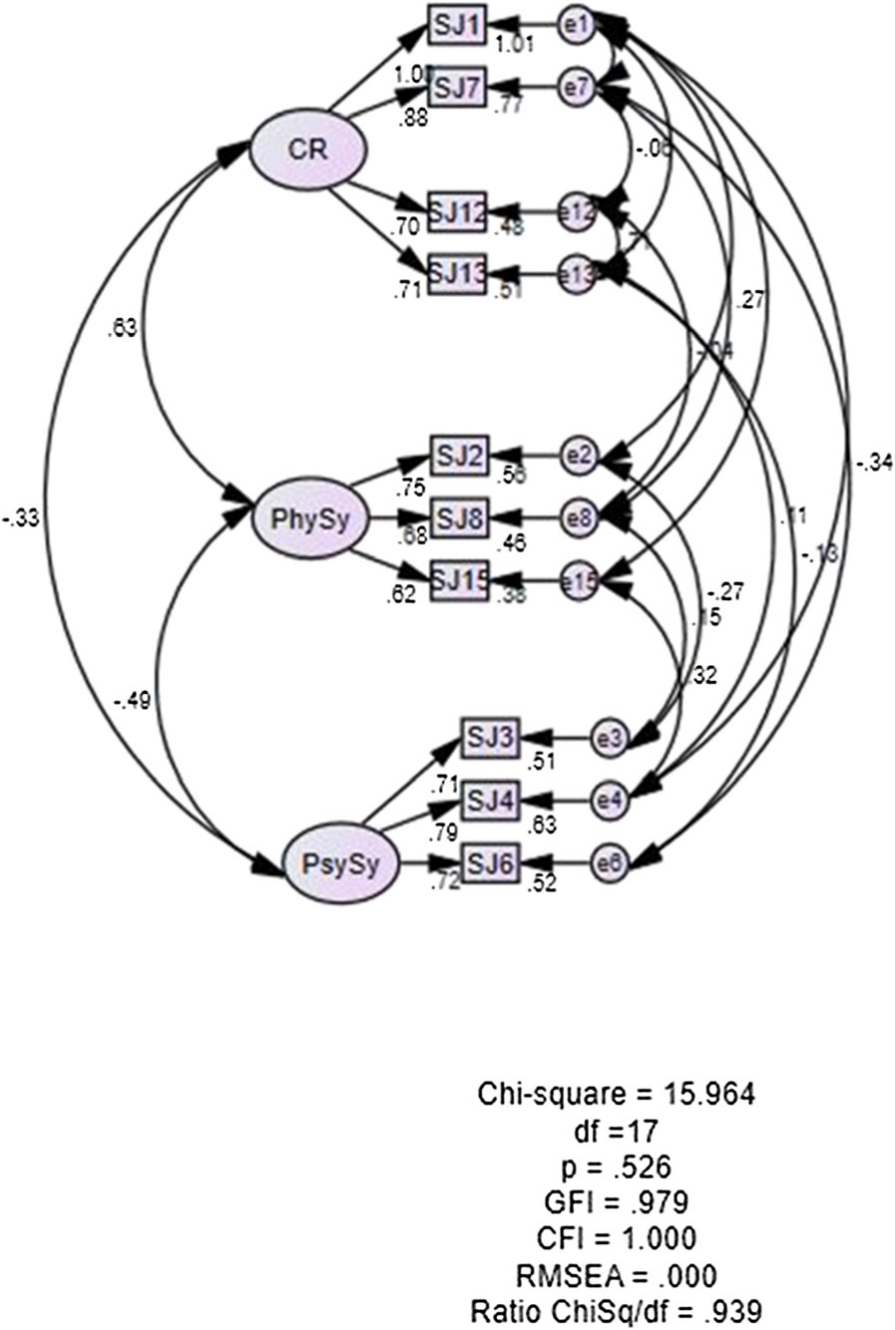
**Structural Equation Modeling for Shiffman-Jarvik Smoking Withdrawal Scale (SJWS-M).**

**Figure 3:**
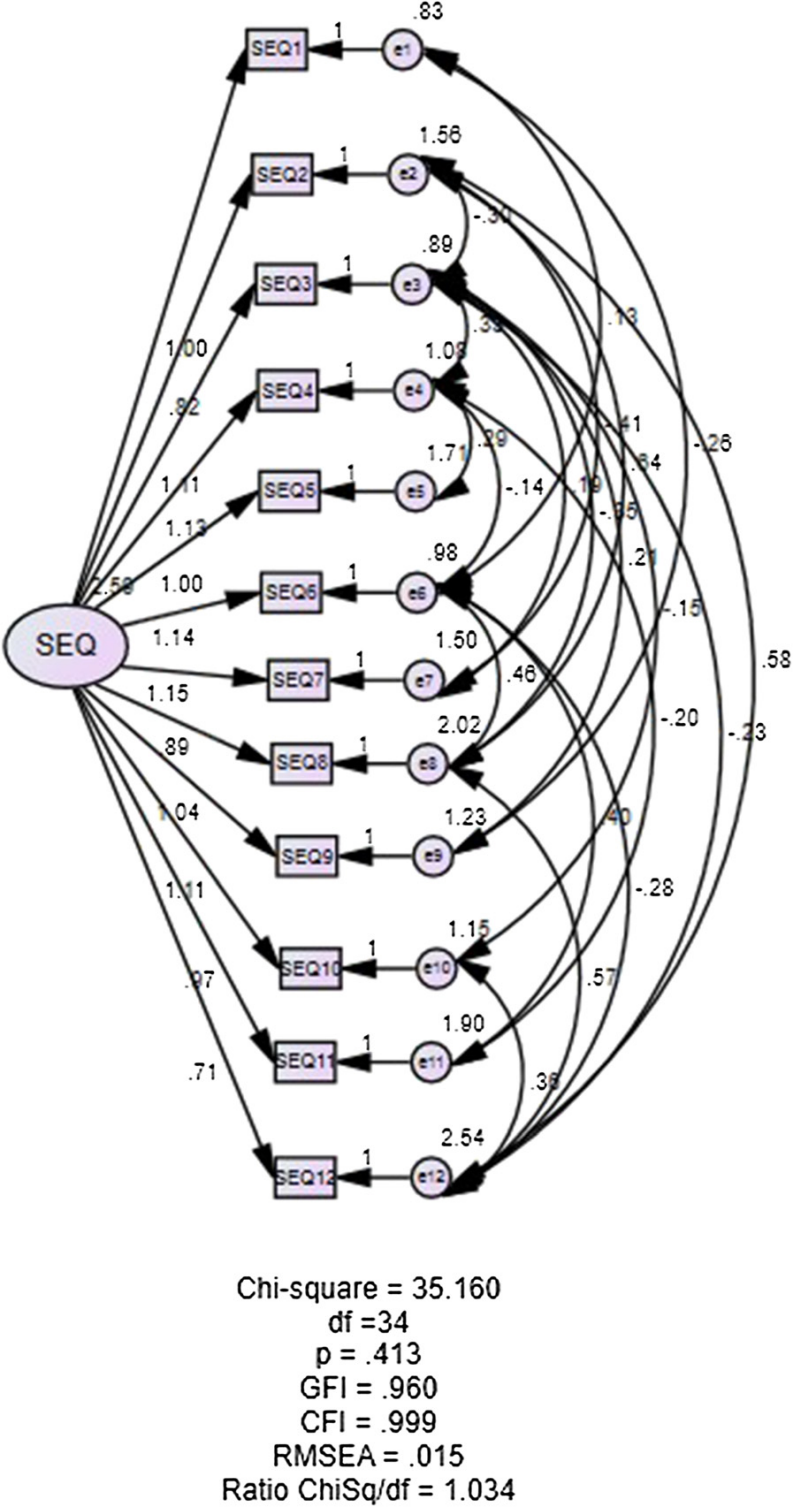
**Structural Equation Modeling for Cessation Self-Efficacy Questionnaire (CSEQ-M).**

Construct validity based on model fit indices showed good model fit. SJWS-M revealed fit data of χ^2^ (df = 17) = 15.964, *p* = 0.526, GFI = 0.979, CFI = 1.000, RMSEA = 0.000, ChiSq/df = 0.939, (Figure 2), whereas the CSEQ-M revealed fit data of χ^2^ (34) =35.16, *p* = 0.413, GFI = 0.960, CFI = 0.999, RMSEA = 0.015, ChiSq/df = 1.034 (Figure 3). Both questionnaires demonstrated good data fit.

Dimensionalities for CSEQ-M (uni-dimension) and SJWS-M (multi-dimension) were evaluated based on the values of SEM factor loadings. After minor model modification, factor loadings for the SJWS-M ranged from 0.62-1.00, while factor loadings for the CSEQ-M ranged from 0.71 to 2.04. The factor loadings of both questionnaires conformed to the recommendation of ≥ 0.50 [56].

## Discussion

In this study, we examined the translation and adaptation of two questionnaires, SJWS and CSEQ from English to Malay. The translated Malay versions were consistent with the English versions. However, we identified one item (Item 8: “While drinking in a bar”) in the CSEQ that was not culturally relevant in the context of Malaysia, and the word “bar” was changed to “party” so that it can be used with the whole population of Malaysia, more than 60% of whom are Muslims. The choice of word may not provide exactly the same meaning, but captures the same kind of experience for the targeted culture [57,58]. The phrase “drinking in a bar” could be deemed as insensitive towards Muslims as it is usually used to refer to the consumption of alcohol in a bar, and such practice is strictly prohibited in Islam. We acknowledge the need to be sensitive to cultural and religious needs in our quest to translate questionnaires. Translated questions/items were modified with words that are able to capture the same kind of experience in our targeted Malaysian culture [57,58].

The SJWS subscales were reduced from five subscales to three subscales. Hair [56] suggested that at least three or four items were needed in a subscale for internal consistency analyses, while two of the subscales in SJWS only had one item each. After removing three other items (Items 10, 11, 14) due to low factor loading values that were considered not significant, SJWS was reduced from 15 items to 10 items while maintaining sufficient representation of the original variables [49]. The three remaining subscales (cravings, physical, and psychological symptoms) demonstrated good internal consistency as the Cronbach’s alphas were above the recommendation of 0.70 [54]. Although the overall Cronbach’s alpha for the SJWS-M was 0.66, this value is within acceptable range, given that all three factor alpha coefficients were well above 0.70 when analyzed separately [55,56]. Cortina proposed that the number of dimensionalities (multi-dimensions) and the number of items could influence the overall Cronbach’s alpha to be lower than its subscale alphas, as seen in the results for SJWS-M which is multidimensional [59]. It is argued that in a multidimensional questionnaire, “items across dimensions were made orthogonal” causing the average alpha for the scale to be smaller (p.102) [59]. Although it is true that alpha is a function of item inter-correlation, it is believed that alpha is also affected by the number of items; lower number of items result in lower alpha, as proven by Cortina [59].

The CSEQ-M was also found to be reliable with good internal consistency. This alpha value is parallel to studies that have used the original English CSEQ that demonstrated Cronbach’s alphas at 0.87 and 0.96 [31,34].

Other than internal consistency, results of the present study indicate that the translated versions of both questionnaires demonstrated good test-retest reliability, discriminant validity, convergent validity, construct validity, and factor loadings.

However, during the face validity tests, recommendations were made to change one of the words in the SJWS-M, Item 11 (i.e., the word “tegang” to the word “stres”, which is a Malay adaptation for the English word “stress.”). Although the word “tegang” is acceptable in the context of physical activity, physical education, sports or fitness and does not threaten the validity of the item, the word “tegang” could also yield double meanings if used in non-sports contexts, such as “strained”, “tight”, “nervous” or “sexually aroused”.

There were two limitations. First, the majority of our participants were males. This could be caused by the disparity of smoking prevalence whereby 43.9% of Malaysian male smokes as compared to only 1% of Malaysian females that smoke. Second, our participants were recruited from an urban population. This could limit the generalizability of our study. Future direction could investigate the construct validity from a theoretical point of view for example if there is any difference between seasoned (long time) smokers versus novice (short term) smokers, whether self-efficacy is lower among seasoned smokers versus novice smokers, and evaluate the usability of these validated SJWS-M and CSEQ-M questionnaires in local smoking research and other Malay speaking countries such as Brunei and Indonesia.

The present study is important because it marked the precedent effort to translate and validate the SJWS and CSEQ into Malay language for the Malaysian population. Through our rigorous translation and validation process, we have presented two reliable and valid questionnaires for future studies on smoking cessation and withdrawal symptoms among local smokers. This is important in light of the recognition that reduction of smokers in Malaysia is an important national health agenda [60]. The experience of withdrawal symptoms is a strong driver of relapse among smokers who attempt to stop smoking [25,61]. Many smokers could feel discouraged to stop smoking due to the anticipated withdrawal symptoms as measured by the SJWS-M. Thus, it is vital to measure withdrawal symptoms in research on smoking cessation, leading to more effective strategies to reduce withdrawal symptoms, such as combining withdrawal with an increase in physical activity [32,62,63] in which the measurement of withdrawal would be a central outcome. Similarly, low self-efficacy to stop smoking is a key reason that many smokers do not attempt to stop smoking. Inclusion of the CSEQ-M in smoking cessation research in Malaysia will help researchers to understand the role of self-efficacy in smoking cessation. This will also lead to strategies to enhance self-efficacy during smoking cessation studies in which the CSEQ will also be a valuable tool.

## Conclusion

The SJWS-M and CSEQ-M are both conceptually and linguistically equivalent to the original English versions. The present psychometric evidence (validity and reliability) confirmed their suitability for future smoking cessation and withdrawal studies in Malaysia.
